# Senescent T Cells in Age-Related Diseases

**DOI:** 10.14336/AD.2024.0219

**Published:** 2024-02-18

**Authors:** Pei-Jie Yu, Mei Zhou, Yan Liu, Jie Du

**Affiliations:** Beijing Anzhen Hospital, Capital Medical University; The Key Laboratory of Remodeling-Related Cardiovascular Diseases, Ministry of Education; Beijing Collaborative Innovative Research Center for Cardiovascular Diseases; Beijing Institute of Heart Lung and Blood Vessel Diseases, Beijing 100029, China

**Keywords:** aging, senescent T cells, age-related diseases, therapeutic targeting, immunosenescence

## Abstract

Age-induced alterations in human immunity are often considered deleterious and are referred to as immunosenescence. The immune system monitors the number of senescent cells in the body, while immunosenescence may represent the initiation of systemic aging. Immune cells, particularly T cells, are the most impacted and involved in age-related immune function deterioration, making older individuals more prone to different age-related diseases. T-cell senescence can impact the effectiveness of immunotherapies that rely on the immune system's function, including vaccines and adoptive T-cell therapies. The research and practice of using senescent T cells as therapeutic targets to intervene in age-related diseases are in their nascent stages. Therefore, in this review, we summarize recent related literature to investigate the characteristics of senescent T cells as well as their formation mechanisms, relationship with various aging-related diseases, and means of intervention. The primary objective of this article is to explore the prospects and possibilities of therapeutically targeting senescent T cells, serving as a valuable resource for the development of immunotherapy and treatment of age-related diseases.

## 1. Introduction

Aging is an irreversible natural process, the prevalence of aging populations is increasing globally every year, sparking increased interest in anti-aging research. The presence of senescent cells in the life cycle can lead to the secretion of various factors that negatively impact tissue function [1]. However, given the difficulty of current interventions in targeting all senescent cells, it is crucial to first identify which cells are the earliest to senesce or can trigger a cascade of aging processes. By identifying the culprits, we can counteract the hazardous impact of senescent cells with maximum efficiency.

The immune system acts as the firewall, protecting the human body from internal and external crises, and is the core force guarding against foreign pathogens and eliminating senescent and tumor cells. Researchers from Niedernhofer Lab decreased the expression of the excision repair cross-complementing group (ERCC)1-XPF endonuclease in the hematopoietic cells of experimental mice and observed disruption of endogenous DNA damage repair, causing the depletion and decline of distinct types of immune cells and impaired immunity. Comparative observations in young and old ERCC1-flox mice revealed that immune cells preferentially undergo senescence. Furthermore, splenocyte transplantation experiments demonstrated that immune cells in a senescent state contribute to the onset of systemic senescence, whereas transplanting young immune cells retards systemic senescence [2]. This finding may provide crucial insights into current research on senescent cells, i.e., that the senescence of immune cells can serve as a starting point of systemic senescence and that targeting the senescence of immune cells will solve aging-related problems.

The immunologic theory of aging, which suggests that immune responses decrease with aging, was first presented in 1964 to describe age-related adjustments occurring in the immune system [3], and the term immunosenescence was coined in 1978 [4]. Thomas and Burnet introduced the concept of immunosurveillance [5-7], whereby the immune system recognizes specific antigens of cancer cells, triggering an immune response mechanism to eliminate them. With aging, immunosurveillance declines, and the body's immune efficacy may subsequently decrease [8].

**Figure 1.**
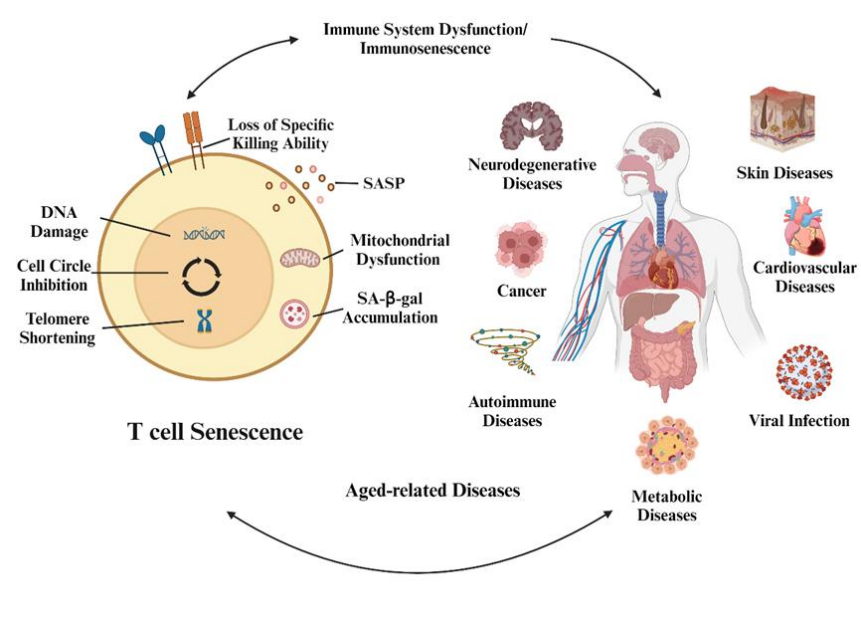
**Relationship between T-cell senescence and aging-related diseases**. Senescence of T cells is the dominant player in immunosenescence, which triggers systemic senescence and contributes to a wide range of diseases. Created with BioRender.com.

Since its discovery, immunosenescence has been widely identified as harmful, potentially leading to low-level, sterile, chronic inflammation known as inflammaging [9]. Immunosenescence and the resulting inflammaging are often associated with age-related diseases [10-12]. Immunosenescence leads to a complex and multifaceted decline in innate and adaptive immune functions. The resulting immune dysfunction is instrumental in most biological modifications that occur in the body during physiological aging and age-related diseases, including cardiovascular diseases [13-16], autoimmune disease [17-20], cancer [21-23], type 2 diabetes [24-26], neurodegenerative disease [27-30], and frailty [10,31]. Moreover, medical treatments that rely on immune function, such as the coronavirus disease 2019 (COVID-19) vaccine, may be less effective in the presence of immunosenescence [32-35]. Therefore, immunosenescence is likely the culprit that triggers aging in the body (Fig. 1).

Alterations in the immune system with aging can alter the number and functions of immune cells, including cell senescence. Although the characteristics of innate immune aging have not been clearly defined [36,37], the senescence of specific cells of the acquired immune response, particularly T cells, has been comprehensively studied. As the main protagonists of cellular immunity, the role of T cells in immunosurveillance is indispensable, including the regulation of senescent cell populations [38]. Previous research has demonstrated that the adaptive immune system, particularly T cells, undergo the most remarkable changes during aging [39]. Even T virtual memory (T_VM_) cells, considered a semi-differentiated cell type, have been found to senesce in humans [40]. T cells are crucial white blood cells, accounting for approximately 70% of lymphocytes and 7-24% of immune cells in the blood [41]. Senescent T cells could contribute to the development of various age-related diseases and influence immunotherapies, for example, vaccines and adoptive T-cell therapies [42,43]. Consequently, understanding and studying T-cell senescence to overcome immunosenescence and advance the treatment of age-related diseases may be of indispensable value in future investigations.

Therefore, this review aims to provide a summary of senescent T cells and their characteristics and recognition markers, mechanisms of formation, and role in various age-related diseases. We are certain that productive immune rejuvenation strategies can contribute to the treatment of age-associated diseases.

## 2. Definition and origin of senescent T cells

Cellular senescence is a condition of permanent cell cycle arrest that occurs when cells exhaust their replicative potential when exposed to stressors but continue to be viable and metabolically functional, accumulating with aging [1,44]. Immune cell senescence research dates back to the late 1970s and was primarily concerned with age-related changes in mouse macrophages and lymphocytes [1,4]. As with immunosenescence, the definition of senescent T cells is continually being updated; T-cell senescence typically refers to the state in which T cells produce inflammatory cytokines but cannot proliferate, triggered by the T-cell receptor (TCR) in response to persistent lifelong antigenic stimulation [45]. Aging-related deterioration of the human thymus and bone marrow can alter the ratios of various T cells, decrease the number of naïve T cells, and significantly reduce CD8^+^ T cells. In contrast, CD4^+^ T cells maintain homeostatic proliferation to preserve their numbers [46]. The quantitative alterations in T cell subsets can reduce TCR clonal diversity, increase memory T cell subsets, and accumulate dysfunctional or exhausted cells, which can negatively impact T cell homeostasis [47,48]. T cells are consistently exposed to immediate and sustained stressors. Commencement of the deoxyribonucleic acid (DNA) damage recognition process, which prevents chromosomal instability and maintains genomic stability, leads to irreversible growth inhibition. Therefore, similar to other cells, T cells inevitably enter senescence and accumulate over time.

## 3. Features of T-cell senescence

Many features are shared between senescent T cells and senescent somatic cells, including fibroblasts. Although no rigorous markers have been identified to specifically identify senescent T cells, extensive genetic and epigenetic studies have been performed, and their characteristic cytokines and surface proteins have been defined. During organismal aging, the gradual development of T cells into a dysfunctional state is marked by disrupted homeostasis and immune-suppression, while the epigenetic remodeling and metabolic reprogramming of these cells can result in senescence or even apoptosis [45,49,50]. Consequently, senescent T cells are markedly inhibited in their expansion and resistant to apoptosis. They simultaneously maintain senescence-associated secretory phenotypes (SASPs), related metabolic changes, and their unique altered killing function.

### 3.1. Senescence-associated beta-galactosidase (SA-βgal) activity

SA-βgal, β-galactosidase detectable in senescent cells at pH 6.0, is a widely used marker in studying cellular senescence hierarchy [51,52]. Senescent T cells, induced by telomere shortening and the p16 pathway, maintain marked SA-βGal activity [53]. Functional analysis of T cells with high SA-βGal activities revealed various enriched gene pathways, including the p53 pathway, inflammatory response, and transforming growth factor beta signaling, many of which exhibit similarities with senescence-induced pathways in human fibroblasts. Furthermore, the differentially expressed genes (DEGs) in T cells with intense SA-βGal levels are largely consistent with those in replicative senescent human fibroblasts [53]. The similarities between senescent T cells and senescent somatic cells are apparent, suggesting a potential link.

### 3.2. Mitochondrial dysfunction and metabolic characterization

Metabolic preferences of T cells are governed by their state of differentiation and mitochondrial function. The signaling function of mitochondria involves the generation, relay, and response to calcium ions or reactive oxygen species (ROS) in cells, contributing to cellular signaling pathways [48]. Increased mitochondrial ROS production in T cells may be correlated to senescence, as suggested by a small cohort study [54]. Although ROS production by mitochondria is essential for T cell activation and regulation, an imbalance between ROS production and accumulation can lead to DNA injury induced by oxidative stress, contributing to the development of senescence and various diseases associated with aging [54].

Specific defects in the induction of single-carbon metabolizing enzymes, as well as impaired respiratory and glycolytic functions, have been illustrated in senescent T cells [55-58]. Physiologically, T cells can utilize oxidative phosphorylation or glycolysis to meet their energy requirements. However, senescent CD8^+^ T cells exhibit severe mitochondrial dysfunction, causing the energy metabolism mode to turn to glycolysis [58]. Mitochondrial dysfunction caused by the deficiency of mitochondrial transcription factor protein A leads to a range of phenotypic conversions of senescent T cells, including metabolic variations and functional alterations. This would, in turn, induce the accumulation of circulating cytokines, resulting in inflammaging that promotes the aging of other cells or tissues in the body and triggers various diseases [55].

Additionally, reduced expression of the glucose transporter protein type 1 (GLUT1) and fatty acid transport proteins (FATP) expressed by senescent T cells leads to impaired nutrient uptake [56], which is consistent with the signs of human aging [59]. After inhibition of p38-mitogen-activated protein kinase (MAPK), restoration of mitochondrial function and enhancement of cell proliferation in senescent T cells occurs; however, the additional cell cycle energy demand does not alter the preference of T cells to obtain energy from glycolysis [57]. The heightened energy demand resulting from p38 inhibition is satisfied by increased T cell autophagy independently of mammalian target of rapamycin (mTOR) complex 1 (mTORC1) [57]. Interestingly, human CD8^+^ T cells showcase a more notable susceptibility to mitochondrial dysfunction than other cells, leading to the rapid acquisition of a senescent phenotype. Meanwhile, senescent CD4^+^ T cells preserve a greater mitochondrial density, take up more lipids and glucose, and use oxidative and glycolytic metabolism. Therefore, the intrinsic diversity between mitochondrial content in different T cell subsets is the primary driver of the metabolic advantage observed in CD4^+^ T cells [56].

Notably, T_VM_ cells accumulating in the bloodstream with age retain the highest mitochondrial capacity among all T cell subsets in aged individuals [40,60]. T_VM_ cells maintain their proliferative capacity during aging through their ability to divide asymmetrically [61]. However, the mechanism underlying the preservation of mitochondrial function remains unclear, perhaps due to the high secretion of interleukin (IL)-15 by T_VM_ [62,63]. Additional research is needed to better characterize T_VM_ cells and their senescent subpopulations to facilitate the possibility of maintaining T-cell mitochondrial function during aging.

T cells also exhibit a specific age-related fading in miRNA (miR)-181a in humans and mice [64], which may be secondary to a decline in the transcription factors Yin-Yang 1 and T cell factor 1. Mature T lymphocytes with miR-181a specific knockdown produce a phenotype similar to that of human T-cell senescence: upregulation of the dual specificity phosphatase 6-sirtuin 1 pathway, reduced T-cell expansion, and impaired viral clearance [65]. In T-cell senescence, the upregulation of aerobic glycolysis was observed in miR-146a^-/-^ T cells, similar to the role of mitochondrial dysfunction, while miR-155 deletion results in reduced glycolysis. Meanwhile, the shortened lifespan of miR-146a^-/-^ mice due to age-related chronic inflammation can be reversed by deleting miR-155 specifically in CD4^+^ T cells [66]. Hence, metabolic reprogramming affects T cell function during aging and certain microRNAs can regulate aging on a molecular level in senescent T cells.

Overall, the metabolic alterations in senescent T cells are primarily associated with mitochondrial dysfunction, for which an inherent difference is observed between CD4^+^ and CD8^+^ T cells, representing a potential direction for targeting applications. The capability of T_VM_ cells to maintain a certain level of mitochondrial function, even in senescent cells, may partially account for the conservation of immune function.

### 3.3. Senescence-associated secretory phenotypes (SASP)

SASP is a complex secretory program transmitted to surrounding cells in a non-cell autonomous fashion associated with senescence onset. Similar to fibroblasts, T cells that enter senescence produce a diverse array of cytokines with inflammatory regulating effects that modulate the microenvironment. The production of IL-6, IL-8, CXC motif chemokine receptor (CXCR), CXCR1, and CXCR2, and metalloprotease domain 28 is remarkably upregulated in senescent T cell subsets compared to naïve T cell subsets [67,68]. In addition, senescent T cells exhibit a significant upregulation of inflammatory factors, including interferon-gamma (IFN-γ) and C-C motif chemokine ligands (CCLs) [69]. The production of a unique SASP dependent on the p38 pathway is observed in senescent T cells [70], indicating a potential impact on the process of immunosenescence and disorders among the aging population. In addition, SASP factors can trigger the senescence of T cells via autocrine or paracrine mechanisms and, subsequently, senescence in other cells [71,72].

### 3.4. Loss of antigen-specific killing function

T cells use the TCR on their surface to identify antigens. In 2014, a study using high-throughput Illumina sequencing demonstrated that the T cell receptor beta chain diversity of human T-cells declines linearly with age and is significantly reduced by 40 years [73]. The signaling activity of the TCR is diminished in senescent cells, and the expression of critical TCR signaling conjugates such as cluster of differentiation 3 (CD3), CD27, and CD28 is also reduced [74,75]. In addition, senescent T cell activation induced by specific antibodies resulted in a remarkable reduction in inflammatory factor and granzyme B production compared with cells in other states [76]. According to these studies, senescent T-cells experience a decline in their antigen-specific killing capacity, which relies on TCR activity.

Nevertheless, senescent CD8^+^ T cells generate a complex that includes the natural killer (NK) receptor NKG2D and NK-articulating molecule DNAX-activating protein of 12kDa (DAP12), which is capable of exerting a certain degree of cytotoxicity on cells expressing the NKG2D ligand [75]. By genetically inhibiting the continued expression of sestrin 2, the expression of NKG2D and DAP12 is reduced while TCR signaling is restored [75]. Therefore, during senescence, targeting sestrins can potentially reprogram senescence-like T cells for broad innate killing activity. This suggests that the specific killing function can be restored, the effects of senescence can be reduced, or senescence can be prevented if the acquisition of the NK capacity of T cells is inhibited.

### 3.5. Changes in epigenetic traits

Substantial epigenetic changes occur in T-cells during aging. Alterations to the epigenome influence the function of genes that encode transcription factors (TFs) and factors secreted during T-cell function. Indeed, the communication between the remodeling of cellular metabolism and epigenetic landscapes is critical for the functional regulation and cell fate decision of T cells during aging [77]. Transcriptomic data from epigenetic assemblies of PBMCs from healthy individuals revealed significant chromatin closing with aging in T cells, leading to the silencing of most genes. CD8^+^ T cells exhibit the most extensive and dramatic chromatin remodeling with aging, with reduced chromatin accessibility leading to decreased transcriptional expression of genes associated with T cell function, including TFs involved in lymphocyte development and activation, such as lymphoid enhancer binding factor 1 and TF 7 (TCF7) [78].

Senescent CD8^+^ T cells exhibit a senescence-associated phenotype with distinct epigenetic programs, featuring chromatin remodeling at specific loci, including increased accessibility at the thymocyte selection-associated high mobility group box and programmed cell death protein 1 loci [78], and a closed chromatin structure at the nuclear respiratory factor 1 locus. Simultaneously, the loss of chromatin accessibility around the CD8^+^ T cell-specific IL-7 receptor (IL7R) locus is accompanied by reduced senescence-associated IL-7R expression. In contrast, in older adults, chromatin closure is observed in genes involved in the IL-7 signaling cascade, such as Janus kinase (JAK)1, JAK3, signal transducer and activator of transcription (STAT)5A, and STAT5B [78,79]. IL-7 is crucial in maintaining T cell expansion and homeostasis and is the most effective cytokine in increasing CD8^+^ T cell cytotoxicity [80-82]. Inhibition of the IL-7 signaling pathways might account for the reduced proliferation and immune responsiveness of CD8^+^ T cells in older adults [83]. Additionally, JAK-STAT signaling pathways hyperactivate in senescent non-immune cells, promoting the secretion of SASP, in contrast to the suppression of STAT in CD8^+^ T cells of older adults. Although SASP components may vary among distinct cell types [84], senescent CD8^+^ T cells display an SASP defined by the expression of IL-18 and CCL-16, which differs from other senescent cells, such as fibroblasts. Moreover, SASP production in senescent T cells might be activated by other pathways, such as p38-MAPK [68]. These studies highlight the unique features of senescent T cells.

DNA methylation regulates chromatin accessibility and gene expression in senescent T cells. A recent study compared genome-wide DNA methylation between senescent (CD28^-^) and non-senescent (CD28^+^) CD4^+^ T cells. The demethylated genes in CD28^-^ T cells are associated with cytotoxicity and cytokine/chemokine signaling, whereas the de novo-methylated genes are enriched in defects in the TCR signaling pathway. This may explain the preactivation state exhibited by CD28^-^ T cells with high expression of inflammasome-related genes. [79].

In conclusion, the epigenetic changes in senescent T cells reflect the relationship between their gene expression tendency and cellular senescence, suggesting the possibility of intervening in T-cell senescence through epigenetic regulation.

### 3.6. Resistance of senescent T cells to apoptosis

Non-immune senescent cells are sufficiently resistant to apoptosis, necessitating the determination of whether the same properties are present in senescent T cells. The diminished apoptotic capacity of senescent T cells can be observed by various stimuli that can trigger apoptosis in non-senescent T cells, such as Fas/CD95 antibodies [85]. Human T cells cultured in vitro are more resistant to apoptosis after senescence induction [86]. Meanwhile, B-cell lymphoma (BCL)-2 expression is upregulated in senescence-induced T cells [87].

Surprisingly, among the elderly, aging T cells may exhibit increased susceptibility to apoptosis. miR-24 overexpression by CD28^-^ T cells leads to DNA damage and is associated with an increased susceptibility of T cells to apoptosis [88]. Even so, IL-15, a cytokine secreted with increased age and highly abundant in the bone marrow, allows CD8^+^ CD28^-^ T cells to upregulate the anti-apoptotic molecule BCL and rescue them from DNA damage-induced apoptosis [88]. Therefore, although T cells of older adults are more susceptible to apoptotic tendencies triggered by stimulation, other factors prevent their apoptosis, ultimately presenting as a reduction in their apoptotic capacity.

Once generated, senescent T cells persist due to their ability to resist apoptosis, leading to their gradual accumulation over time. Therefore, the apoptosis reduction of senescent T cells is predicted to lead to their gradual occupation of an increasing pool of memory T cells in older adults, limiting the reserve of remaining T cells.

### 3.7. Surface markers

Senescent T cells exhibit a distinct phenotype characterized by the loss of co-stimulatory receptor CD27/CD28, upregulation of CD57, CD45RA, and killer cell lectin-like receptor G1 (KLRG-1), and downregulation of C-C motif chemokine receptor (CCR)7 and CD45RO, suggesting that senescent T cells also exhibit a terminal differentiation phenotype, which is distinct from other terminally differentiated T cells [49,89-92]. Enhanced KLRG-1 expression, an inhibitory receptor on T cells that transmits negative signals to modulate the immune response, may contribute to the reduced proliferative capacity of senescent T cells, as evidenced by the increased AKT (ser473) phosphorylation and cell growth and division upon TCR stimulation following KLRG-1 inhibition [93]. This suggests that functional defects in T-cell senescence may be attributable to inhibitory receptor signaling [93,94]. In addition, the recognition and identification of senescent T cells in humans and mice have been facilitated by immune checkpoint receptors, such as immunoglobulin-like transcript 2 (ILT2), or by markers such as FAS/CD95 and CD153 [75,95-98].

T cells in older adults that express T cell immunoreceptors with immunoglobulin and immunoreceptor tyrosine inhibitory motif structural domains (TIGIT) exhibit high levels of inhibitory receptors. Functional deficits associated with senescence in this cell population are reversed via TIGIT knockdown [76]. Although certain markers, such as programmed cell death protein 1 (PD-1) and lymphocyte activating 3, are also present on senescent T cells [99], these markers are frequently observed on T cells that have undergone exhaustion, a state of functional impairment that arises from chronic antigen exposure and are not to be singled out as senescence markers to differentiate between the two. The features of senescent T cells can change depending on the circumstances; therefore, accurately tracking senescent T cells by combining functional and surface markers to provide a solid theoretical basis for the precise identification and targeting of senescent T cells is desirable.

As mentioned earlier, irreversible growth arrest is the feather of senescent cells. Notably, the scenarios regarding restoring the proliferative capacity of senescent cells result from artificial, experimental interventions and are not representative of the natural aging process.

## 4. Formation of senescent T cells

**Figure 2.**
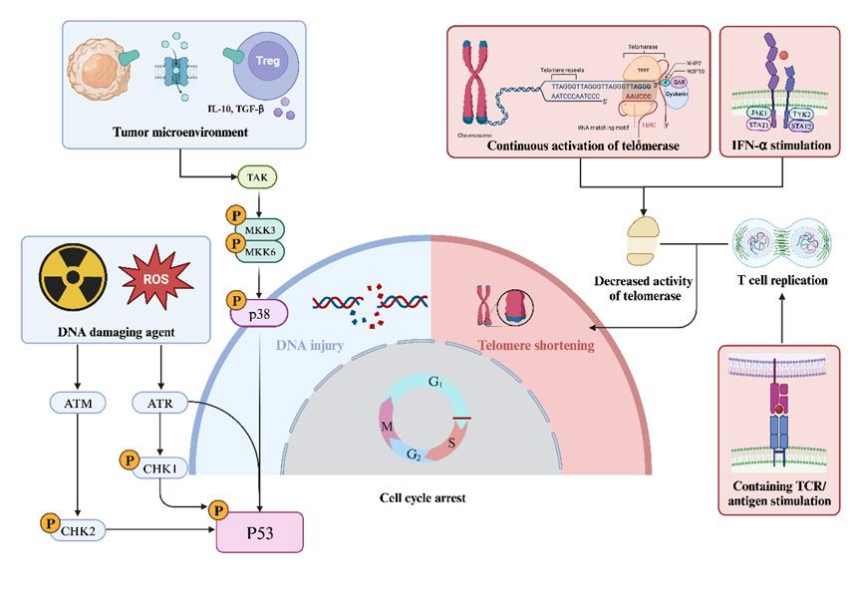
**Information of T cell senescence**. Replicative senescence of T cells is due to stimulation by TCR/antigen, which constantly divides and replicates to meet the immune demand, causing the continuous shortening of telomeres and cell cycle stagnation. However, the activity of telomerase is affected by some of the factors or decreases in the process of continuous telomere repair, ultimately losing the repair function. The stress-induced T cell senescence is triggered primarily by the stimulation of exterior aspects, such as tumor cells and T regulatory (Treg) cells in a tumor microenvironment, through metabolic competition-induced T cell DNA damage by the TAK/MKKs/p38 pathway, forcing T cells into senescence. Other DNA damage-inducing factors, such as ROS and IR, through the P53 pathway can cause T cell senescence in vitro. Created with BioRender.com.

Influencing factors of cellular senescence continue to be reported, improving our understanding of the mechanisms underlying cellular senescence. Cellular senescence was first identified as limited proliferation in normal somatic cells after repeated passages in culture, designated replicative senescence [100]. However, as new mechanisms of senescence continue to be reported, cellular senescence is more likely attributed to DNA damage than replication-induced telomere shortening [101]. Similar to most cellular senescence, cell cycle-associated INK family inhibitors (e.g., p16INK4A) reduce the phosphorylation of retinoblastoma tumor suppressor protein by inhibiting cell cycle protein-dependent kinases (CDK)4 and CDK6, inhibiting T cell cycle progression from G1 to S phase [102-105]. The following section discusses telomere-dependent senescence of T cells and cellular senescence caused by other pathways, as summarized in Figure 2.

### 4.1. Replicative senescence

Telomeres are highly repetitive DNA sequences located at the end of eukaryotic chromosomes, and their key role is to stabilize the structure of chromosomes and control the cell division cycle. Telomere shortening is caused by ongoing cell division and decreased telomerase activity. It is essential to maintain the integrity of telomeres to delay aging.

Telomere shortening with aging has been observed in human T cells and other non-immune cells. Repeated stimulation by antigens can maintain continuous proliferation of antigen-specific T cells in vivo; proper activation of T cell receptors and sustained exposure to IL-2 are necessary for the survival of normal human T cells in vitro, with cultures undergoing approximately 33 generations of cell replication before reaching the end of the replicative lifespan and stopping proliferation [106,107]. Several factors, including tumor necrosis factor-alpha (TNF-α), directly inhibit telomerase activity and accelerate aging [108]. Additionally, deleting CD27/28 may reduce the expression of human telomerase RNA component (hTERC), resulting in decreased telomerase activity. However, T cells possess high telomerase activity during developmental stages and after mature T cell activation, which differs considerably from non-immune cells. Therefore, telomerase can compensate for the loss of lymphocyte telomeres during division [109]. The association between CD27/28 expression and hTERC indicates that deleting these surface markers may reduce telomerase activity. In a recent study, T cells from the spleens of mice were continuously transferred to new young mice and continuously expanded over ten years. This study suggests an unusual replicative potential for mouse T cells. However, this requires young bodies, and the telomeres of mouse T cells are twice as long as those of human T cells; therefore, the existence of this possibility in humans is unclear.

Interestingly, CD8^+^ T cells present a distinct pattern of replicative senescence compared to CD4^+^ T cells. The most reliable identifier of in vivo CD8^+^ T cell senescence is the absence of CD28 expression. Following multiple rounds of CD8^+^ T cell activation, the downregulation of CD28 due to inactivation of the corresponding TFs occurs with subsequent reduction of CD27. CD4^+^ T cells senesce at a lower rate than CD8^+^ T cells, accumulating oligoclones slowly [74,92,110].

### 4.2. Stress-induced cellular senescence

Ongoing research on senescent cells has revealed that cellular stresses, such as ROS, chemotherapy, radiation, and metabolites, may play a major role in cellular senescence. Oxidative stress increases with age due to mitochondrial dysfunction and the accumulation of ROS, which ultimately leads to DNA damage and cellular senescence [54,111-113]. T-cell senescence can also be triggered by stress. Iterative or excessive TCR stimulation can cause a gradual change in downstream T cell telomerase activity, telomere erosion, irreparable DNA damage, and ultimately, dysfunctional replicative ability when T cells re-encounter the antigen, causing senescence [74,114-116]. Cyclic adenosine monophosphate derived from tumor cells triggers the senescence of naïve and effector T cells [117]. Tregs can cause DNA damage in T cells during crosstalk via glucose competition [118,119]. DNA damage or glucose deprivation is sensed by adenosine monophosphate-activated protein kinase (AMPK), which elicits MAPK p38 recruitment to the transforming growth factor beta-activated kinase 1-binding protein 1 (TAB1), leading to the auto-phosphorylation of p38 and senescence of T cells [74]. By inhibiting the p38 pathway or silencing AMPK and TAB1, the IL-2 and anti-CD3-stimulated proliferation capacity of CD27-CD28-CD4+ T cells is restored [74]. Exposure to DNA-damaging agents, including ROS and ionizing radiation (IR), can also trigger premature senescence by activating stress pathways, such as the p53 pathway, when growth factors are deprived [120]. Stress-inducible proteins sestrins dramatically increase the activation of extracellular signal-regulated kinase (ERK), c- Jun N-terminal kinase (JNK), and p38 pathway. Inhibition of sestrins restores the T-cell antigen-specific and vaccine responses [114,121].

In the case of viral infections, an increased abundance of CD4^+^CD27^-^CD28^+^ cells may be an early indicator of the prioritized loss of CD27 in CD4^+^ T cells, with the eventual loss of the CD28 antigen as cells progress to a differentiated state, in contrast with CD8^+^ T cells [122,123].

In summary, T-cell senescence may arise from integrated cellular stress-induced and replicative senescence. Senescence of T cells may result from repetitive clonal expansion and antigenic recall, reinforcing the immune response against the challenging pathogen.

## 5. Relationship between T-cell senescence and immunosurveillance

Apoptosis is the heart of the specific immunity triggered by T cells in response to virus-infected or pathogenic cells, leading to their elimination [124]. Immune cytotoxicity against various diseases depends on the function of perforin 1 (PRF1), a membrane-permeabilizing protein that forms pores in target cells [125-128]. After knocking out the gene encoding PRF1, apoptosis mediated by granule cytotoxicity is disabled, leading to impaired immune surveillance [129]. Moreover, the killing capacity of senescent CD8^+^ T cells becomes defective following perforin and granzyme loss or defective granule extravasation [130,131]. This suggests that the decline in immune surveillance is largely due to T-cell dysfunction, highlighting the importance of maintaining T-cell function and preventing senescence. In turn, immunosenescence or reduced immunosurveillance leads to increased senescent cells, accelerating the aging process and driving T cells toward senescence [129]. That is, T-cell senescence is a part of immunosenescence, with organismal senescence mutually affecting and even causally related to decreased immune function.

## 6. Role of senescent T cells in different diseases

Senescent T cells have been extensively investigated in numerous infectious and age-related diseases, including COVID-19, cardiovascular, metabolic, skin, cancer, neurodegenerative, and autoimmune diseases. This section discusses the current research hotspots and progress in senescent T cells in various diseases.

### 6.1. COVID-19

COVID-19 has attracted unprecedented global attention and research boom, with several studies providing evidence that aging is a significant influencing factor in the association between COVID-19 and severe symptoms and complications. Meanwhile, among young and older individuals, the aging process is accelerated in patients with neocoronavirus, as evidenced by considerably faster rates of epigenetic aging and telomere depletion compared to healthy individuals; therefore, epigenetic changes in patients with COVID-19 may contribute to post-neocoronasal virus infection syndrome [132]. Low-grade inflammation (inflammaging) induced by immune-senescence may lead to increased COVID-19 severity [133]. Compared to healthy controls, patients with COVID-19 exhibit a higher proportion of T cells expressing cell exhaustion/senescence-related markers, such as PD-1 and CD57, and more activated cells expressing human leukocyte antigen-DR isotype and CD38. Patients exhibit significantly increased inflammation-related factors (like chemokines and galactoglucagon), and their lymphocytes produce more inflammatory factors [134]. Moreover, T helper 2 cell differentiation may occur during the initial phases of COVID-19, along with functional failure or senescence of T cells due to TCR hyperreactivity [135].

Although studying T-cell senescence in COVID-19 is in its infancy, the evidence presented implies a novel therapeutic tactic for COVID-19 by preventing the specific secretion from senescent T cells.

### 6.2. Cardiovascular diseases

Recent researches have reported that senescent T cells contribute to the initiation and development of cardiovascular diseases. The existence of metabolic risk factors for the cardiovascular system in the aging population is linked to an augmented expression of inflammatory factors produced by CD4^+^ T cells, as well as the features of T-cell senescence [136]. Through transcriptome sequencing, researchers have identified T cell subpopulations with senescent features in human atherosclerotic plaques [137].

Circulating senescent T cells may also serve as markers to predict the risk of cardiovascular events. Reportedly, CD8^+^CD57^+^ senescent T cells with extensive IFNγ production accumulate in acute coronary syndrome (ACS) and stable angina (SA) patients [136,138]. Meanwhile, markedly more CD4^+^CD28^-^ senescent T cells have been reported in patients with unstable angina than in SA patients [139]. Furthermore, the increased abundance of CD4+CD57^+^ senescent T cells in patients with acute heart failure predicts adverse clinical outcomes [140].

Mechanistic studies on cardiovascular diseases using senescent T cells are underway in mice. Age-related T cells in cardiovascular diseases have a causal pathogenic effect. Mice with premature T-cell senescence exhibit age-related alterations in the heart, including aortic dilatation, cardiac structure, and myocardial dysfunction [55]. In a hypertension model induced by angiotensin II, the inoculation of T cells from aged mice into young hosts accelerated cardiorenal injury by increasing IFNγ secretion, promoting inflammation and fibrosis [141]. When nonsenescent CD4^+^ T cells are transplanted from healthy humans into young lymphocyte-deficient mice, they experience homeostatic expansion and normal differentiation into a phenotype typically observed in older adults. In addition, the differentiated CD4^+^ T cells infiltrate the heart, leading to myocardial inflammaging [142].

These studies indicate that circulating senescent T cells directly participate in the pathogenesis of cardiovascular disease and present a strong correlation with patient prognosis. Meanwhile, delaying T-cell senescence or removing senescent T cells may be novel therapeutic approaches for cardiovascular disease. However, our understanding of the role of T cells in cardiac homeostasis remains inadequate under physiological and pathological conditions. Hence, further studies are needed to address this issue before investigating how to target T-cell senescence best to intervene in cardiovascular dysfunction.

### 6.3. Metabolic diseases

Immunosenescence-induced inflammaging is a metabolic disease; the involvement of senescent T cells in metabolic disease has drawn significant attention. In humans, the incidence of hyperglycemia is related to the presence of elevated amounts of senescent T cells in the systemic circulation [25]. Patients with type 2 diabetes exhibit significant escalation in functionally impaired senescent T cells (CD45RA^+^CCR7^-^) in their circulation, as well as senescent CD28^-^ T cells in their livers, which are positively associated with fasting glucose levels [143].

Recent research has focused on obesity, where obese mouse models exhibit an enrichment of a unique population of senescence-associated T cells (SA-T cells) in their visceral adipose tissue (VAT). The strong activation of secreted phosphoprotein 1 by this population of senescent T cells leads to inflammation in the VAT. Unexpectedly, when SA-T cells were transplanted into the VAT of non-obese mice, VAT inflammation and insulin resistance occurred. In reverse, eliminating SA-T cells of obese mice enhances glucose tolerance and insulin sensitivity [96,98]. Researchers also identified increased numbers of senescent CD153^+^ T cells in the livers of older mice, which correlated with elevated blood glucose and insulin levels [144].

Collectively, the incidence of common metabolic diseases is associated with the presence of senescent T cells. Additionally, the preliminary validation of the mechanism of some of these senescent T cells in metabolic diseases, especially obesity, has been confirmed in mice. However, SA-T cells do not represent all senescent T cells, and the potential contributions of other senescent T cells to the development of metabolic diseases in humans remain unclear and require further investigation.

### 6.4. Skin diseases

Skin senescence occurs with age or exposure to environmental aggressors (e.g., ultraviolet radiation) and can transmit the senescence phenotype from the skin to other tissues and organs via the SASP. Although skin senescence is a distinct feature of aging that has been actively studied, research on the association between senescent T cells and skin diseases is lacking, and progress on this topic appears to have stalled.

Senescent cells mediate the dermatopathology of human cutaneous leishmaniasis (CL). A fraction of senescent CD8^+^ T cells that accumulate in patients with CL express cutaneous lymphocyte-associated antigen (CLA) in response to the protozoan. In fact, senescent CD8^+^ T cells preferentially accumulate in patients with skin damage because high CLA expression increases the skin-homing potential [145,146]. However, increased CLA-expressing T cells, which possess senescent characterization during CL, may be due to extensive TCR activation and stimulation by Brasilia antigenic epitopes [147,148].

The categories of skin diseases are broad and intersect with autoimmune diseases in particular. Therefore, various age-related skin diseases should represent an immunosenescence research focus area in the future.

### 6.5. Cancer

Immunosenescence impacts the three phases (clearance, homeostasis, and tumor escape) of the interplay between the human immune system and tumors. Despite the intricate and unpredictable role of senescence in tumors, T-cell senescence has been thoroughly investigated in cancer and considerable evidence exists regarding the clinical relevance and mechanisms by which T-cell senescence triggers cancer or vice versa.

In tumors, malignant tumor and Treg cells can induce T-cell senescence [149]. The circulatory system in patients with solid tumors and hematological malignancies contains an accumulation of senescent T cells [25,150-156], the abundance of which increases as clinical staging progresses [157]. After tumor resection or long-term remission, the quantity of senescent T cells typically decreases [154]. Senescent T cells may act as a biomarker to predict clinical outcomes in cancer patients. Many oncological diseases are characterized by shorter overall survival of patients with increased levels of senescent T cells in their bloodstream [158-161]. CD8^+^CD57^+^ senescent T cells also have the ability to forecast the growth of cutaneous squamous cell carcinoma in a portion of patients after renal transplantation [162].

Senescent T cells induced by tumors may also stimulate the production of pro-inflammatory and angiogenic factors by monocytes/macrophages, which can facilitate microtubulogenesis and tumor cell survival [163,164]. For example, induction of immunosuppressive factors, including indoleamine 2,3-dioxygenase, programmed death ligand 1 (PD-L1), and cytotoxic T lymphocyte-associated protein 4, by increased IFN-γ production without granzyme B expression can suppress the toxicity and expansion of T cells [165,166]. This may represent a mechanism of tumor immune escape.

The utilization of senescence has become a prevalent subject in cancer treatment research [167]. However, both senescence induction and senescent cell removal from tumor tissues must be supported by further studies. These results suggest that senescent T cells with suppressive functions or tumor-induced senescence may lead to tumor immune evasion, and delaying or preventing antigen-specific T-cell senescence may become a novel approach for enhancing anti-tumor immunity during aging.

### 6.6. Neurodegenerative diseases

Aging is a significant risk factor for most neurodegenerative diseases. The association between immunosenescence, inflammation, and neurodegenerative diseases is widely recognized, while the connection between T-cell senescence and these diseases is still being explored. For example, T cells in patients with Alzheimer's disease (AD) have shorter telomeres, which is directly linked to elevated circulating TNF-α expression, senescent CD8^+^ T cell occurrence, and increased susceptibility to T-cell apoptosis [168]. Meanwhile, another study found that patients with AD have an elevated proportion of senescent CD4^+^ T cells compared to age-matched controls, with no change in the CD8^+^ T cell phenotype [169]. Although there is no direct support in the literature, the presence of senescent T cells may lead to widespread inflammation throughout the body, causing an increased neurodegenerative disease load.

Research on the correlation between T-cell senescence markers and indicators of Parkinson's disease (PD) progression is lacking; however, PD cohorts at different disease stages have shown comparable reductions in terminally differentiated T cells [170]. Evidence indicates that the impact of age-related immune dysregulation may not be sufficient for disease development but may contribute to its pathogenesis. In one study, compared to controls, patients with PD have a significantly lower proportion of senescent CD8^+^ T cells with no significant change in the CD4^+^ T cell subset [30,171]. Another study demonstrated that in patients with PD the CD8^+^ T cell population was less prone to senescence [172]. Hence, the senescence of different T cell clusters in patients with AD, PD, or other neurodegenerative diseases must be considered in a dichotomous manner, and the mechanisms that lead to the decreased senescence of CD8^+^ T cells in PD remain unclear, warranting further investigation.

### 6.7. Autoimmune diseases

T cell senescence contributes to the development of autoimmunity and chronic inflammation [173]. While most previous studies have focused on understanding the mechanism behind T cell senescence in illness, current research is directed toward identifying a suitable intervention method for T cell senescence with the objective of ameliorating or suppressing the symptoms of diseases like rheumatoid arthritis (RA) and lupus erythematosus.

Patients with autoimmune diseases show a marked increase in the proportion of senescent T cells [174-177], representing a crucial factor in the severity of certain typical diseases [178-180]. The migration of senescent T cells from the blood to sites of inflammation in RA and multiple sclerosis (MS) has been demonstrated in several studies [181,182]. Cytomegalovirus (CMV)-induced T-cell senescence is well described and has been implicated in autoimmune diseases. CMV infection induces the accumulation of expanding overstimulated T cells, which may contribute to immunosenescence. Additionally, a rising number of senescent T cells was observed in patients with RA, MS, and type 1 diabetes due to the persistence of CMV [183-185].

In mice, senescent T follicular helper cells contribute to the development of the disease of lupus nephritis by secreting osteoblastin [186]. The reciprocal activation of SA-T is influenced by the molecular interaction between CD153 and CD30, which spontaneously develop germinal center B cells, introducing an immunosenescent phenotype and autoimmune dysregulation. Blocking the interaction with CD30 using an anti-CD153 antibody inhibits the age-related augmentation in senescence-like T cells and ameliorates lupus symptoms in lupus-prone mice [97].

The age-related elevation in senescence-like T cell levels in cartilage tissues of RA patients is caused by low meiotic recombination 11 homolog A (MRE11A) expression in CD4^+^ T cells, and overexpression of MRE11A can repair this damage [187]. Consequently, slowing the process of immunosenescence in T cells may be advantageous for treating RA.

## 7. Impact of T cell senescence on immunotherapy

Immunotherapy is a medical treatment that enhances immune cells to combat diseases, such as cancer, which has demonstrated promising outcomes in patients [149] with autoimmune disease, cardiac fibrosis, and aging [188-190].

Effective vaccine responses depend on the synergistic action of adaptive and innate immune systems affected by immunosenescence [116]. Although direct evidence that T-cell senescence affects vaccine responses is lacking, a decline in adaptive immune function caused by T-cell senescence can be predicted, further attenuating vaccine action.

In the case of CAR-T therapy, T cells inevitably undergo senescence before the transduction of chimeric antigen receptor (CAR) structures and when CAR-T cells are expanded within the recipient. Compared with younger donors, most T cells from older donors exhibit a CD45RA^+^CD45RO^+^ phenotype, suggesting a shift to memory cells from naïve cells. Such cells moving toward senescence have a restricted capacity for proliferation and make a negligible contribution to the development of the ultimate CAR-T cells. Even at the production stage, their SASP and physical interactions can affect the properties of other T cells, potentially influencing the efficacy of the therapeutic efficiency [43]. Additionally, when T cells are extracted from patients treated with chemotherapy and transduced with CAR structures, the processed CAR-T cells undergo senescence [191]. Other studies have confirmed that pretreatment with cytotoxic drugs, such as cyclophosphamide/adriamycin-containing regimens, is linked to poor CAR-T cell performance [192]. That is, remarkable growth in the quantity of hyperdifferentiated and senescent T cells after chemotherapy treatment or in older patients can lead to decreased transduction and expansion in vitro, reducing therapeutic efficacy. Thus, patients receiving combination therapy with CAR-T and other drugs should be mindful of the effect of drugs on CAR-T cells in terms of senescence. Therefore, avoiding the senescence of T cells, which are critical for the immune process, may be a key issue for future immunotherapy.

## 8. Current drugs or treatments available to target T-cell senescence

**Figure 3.**
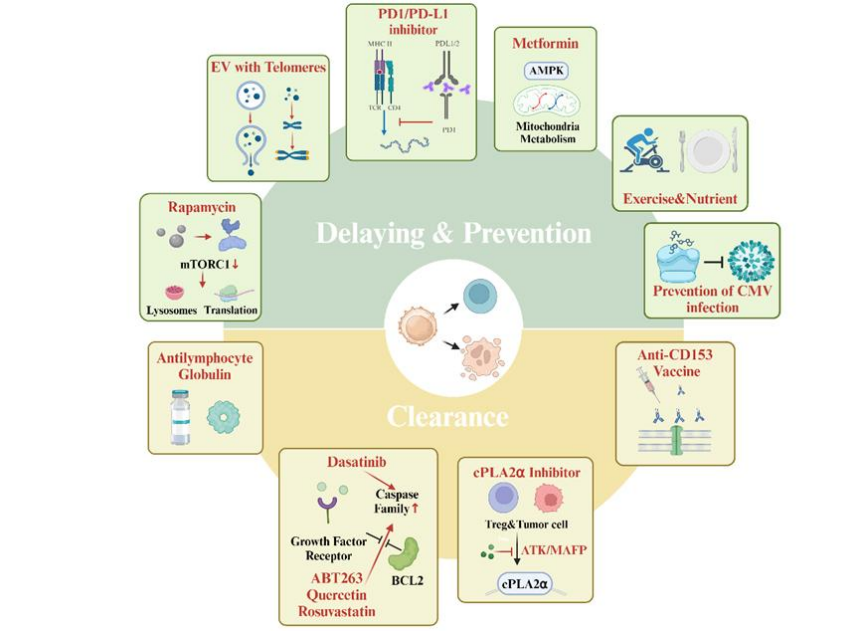
**Current interventions for T cell senescence**. For T cells that are naïve/undifferentiated and others that are not yet senescent, senescence can be slowed by inhibiting the mTOR pathway through rapamycin, promoting autophagy, and reducing protein translation. T-cell activity and mitochondrial metabolism can also be improved by AMPK activators (e.g., metformin), which cause T-cells to have lengthened telomeres, ultimately increasing the chances of replication. In addition, whether PD1/PD-L1 inhibitors are beneficial for treating T-cell senescence requires exploration. Moreover, the exploitation of telomere transfer mechanisms warrants further investigation. For senescent T cells, anti-lymphocyte globulin (ATG) vaccines targeting CD153, and senolytics, such as dasatinib, can promote senescent T cell autophagy or lysis, clearing senescent cells; whereas cytosolic phospholipase A_2_ (cPLA2)α inhibitors can reverse the lipid metabolism disorder of senescent T cells, restoring a certain degree of normal T cell function. Additionally, adequate physical exercise, nutritional intake, and prevention of CMV infection can effectively prevent and alleviate T-cell senescence. Created with BioRender.com.

In the field of aging science, partial reprogramming to reverse aging is currently a popular approach, and repair after aging damage may become a mainstream concept in the anti-aging field. Currently, methods to specifically delay T-cell senescence or remove senescent T cells remain in the research phase, with no widely applicable clinical treatments. However, certain studies have suggested that lifestyle changes, drug therapy, and genetic engineering may help reverse or minimize the effects of senescent T cells. Research to extend the T-cell lifespan and boost the potency of immunotherapy or anticancer therapy is currently a topic of interest for scientists in terms of therapeutic approaches targeting T-cells (Fig. 3).

### 8.1. Antithymocyte globulin (ATG)

ATG is a medication used to directly eliminate senescent T cells. For many years, ATG has been employed as an immune system suppressor to prevent graft-versus-host disease in transplant patients and is capable of eliminating senescent T cells in the human circulatory system [193]. Nevertheless, the study did not address the mechanisms associated with the greater toxicity of ATG against senescent T cells and the prolonged impact of this medication treatment, nor did it discuss its detrimental effects on CD28^+^ T cells.

### 8.2. Senescent cell scavengers (senolytics)

Senolytics are a class of drugs or therapeutics used to remove or reduce senescent cells in the body, with the goal of improving health and prolonging life. These drugs accomplish this by interfering with the survival mechanisms of senescent cells or promoting their apoptosis (cell death). Senolytic research focuses on drug discovery and development, with several drug candidates currently in clinical trials.

#### 8.2.1 BCL-2 inhibitor

ABT-263 became the first reported anti-aging drug with general applicability that specifically removes senescent cells and rejuvenates senescent tissue stem cells. A team of researchers safely used ABT-263 to successfully reduce cell viability and induce apoptosis in senescent T-cell malignancies [87]. Additionally, statins downregulate BCL-2 expression to induce senescent T-cell apoptosis. In patients with acute coronary syndromes, the use of rosuvastatin increases apoptosis in senescent T cells to significantly reduce the proportion of senescent cells, IFN-γ production, and BCL-2 expression [194].

#### 8.2.2 Dasatinib and Quercetin

Dasatinib is a multi-targeted inhibitor of tyrosine kinases that interferes with ephrin-dependent apoptosis inhibition, previously used only in cancer therapy [195,196]. The use of dasatinib in treating patients with atherosclerosis reduces senescent circulating T follicular helper cells and senescent gene expression, demonstrating its role in the incomplete elimination or restoration of these cells. This results in the disposal of inflammatory mediators and decelerated disease advancement [197]. However, evidence supporting the opposing outcome has also been reported; leukemia patients' T-cell counts also increase during dasatinib treatment, and their phenotype tends toward senescence [198]. Additionally, dasatinib can stop the cytolytic activity and proliferation of CAR-T cells in vitro and in vivo, while its discontinuation results in rapid and complete restoration of CAR-T cell function. Thus, dasatinib might act as a switch for CAR-T cell therapy [199].

Quercetin is a naturally occurring flavonoid found in many fruits and vegetables with anti-aging properties [200]. The combination of two anti-aging drugs, D and Q, significantly eliminates senescent cells and slows age-related disease progression. Moreover, in an influenza mice model, it led to improved Treg differentiation in lungs, while significantly reducing transforming growth factor beta levels in bronchoalveolar lavage fluid [201]. Nevertheless, there is no clear evidence of a combination therapy for the removal of other senescent T cells. This is partly because quercetin curtails T cell differentiation and proliferation by inhibiting the quantity of IL-2 and IFN-γ produced by T cells, thereby suppressing their activity [202,203].

#### 8.2.3 Immunotherapy removes senescent cells

The introduction of anti-aging vaccines has led to a novel approach for removing senescent cells, which essentially involves generating specific immune memory for specific senescent cells through the autoimmune system. In 2020, a team successfully manufactured a CD153 antigen vaccine that prevents the buildup of senescent CD153+ T-cells in mouse adipose tissue and improves parameters associated with obesity, glucose tolerance, and insulin resistance [96].

### 8.3. Regulation of the mTOR pathway

Studies have revealed that rapamycin, a potent inhibitor of the mTOR signaling pathway, is highly effective in extending the lifespan of mice and shows promise in treating various aging-related diseases. mTOR inhibitors limit protein synthesis and may restore cellular autophagy while reducing translation frequency [204]. Targeting the mTOR pathway for immune enhancement in older individuals has shown promising results, and the involvement of the tumor suppressor protein menin in preventing T cell senescence through mTORC1-dependent metabolic activation is being explored [205-207]. In CAR-T cells cultured in vitro, rapamycin and IL-15 act similarly in that both inhibit T cell transformation to a hyperdifferentiated or senescent phenotype and maintain the memory phenotype of poorly differentiated stem cells, as defined by CD62L^+^CD45RA^+^CCR7^+^ expression [208].

### 8.4. Regulation of the AMPK

The AMPK pathway appears to be associated with cellular senescence. Previous research has demonstrated that AMPK activators can enhance the activation and mitochondrial metabolism of certain T cells. By enhancing cellular autophagy and improving mitochondrial energy cycling, metformin ameliorates inflammatory responses in T-helper (Th)17 cells [209]. Meanwhile, metformin inhibits nicotinamide adenine dinucleotide phosphate oxidase 4 and reduces non-mitochondrial ROS production in senescent cells, preventing the development of radiation-induced weakness and disability in senescent mice [210]. A recent study has shown that in vitro treatment of PBMCs from a middle-aged population with metformin significantly reduces the number of senescent T cells, inhibits the secretion of IFN-γ and IL-6 by senescent T cells, and induces an increase in telomerase concentration and frequency of undifferentiated T cells [211].

### 8.5. PD-1/PD-L1 inhibitor

Inhibitory receptor signaling, like PD-1, can be blocked to improve T cell multiplication and function. For example, berberine (BBR) promotes the degradation of PD-L1 through a ubiquitin pathway [212], improves immunosuppression of T follicular helper cells [213], and activates factors produced by Tregs [214]. Moreover, senescence surveillance is enhanced by blocking this checkpoint, thereby reducing the occurrence of senescent cell immune escape [215]. Thus, although PD-1 is an indicator of exhaustion in most T cell cases, its relationship with T cell senescence warrants further investigation.

### 8.6. Regulation of the MAPK pathway and STAT pathway

MAPK significantly influences the supervision of T-cell senescence. According to the results of preclinical experiments, inhibitors or activators targeting p38, ERK, JNK, and STAT signaling can inhibit T-cell senescence [57,68,74,114,119,216,217]. The JAK-STAT signaling pathway also plays an important role in regulating T-cell senescence. Meanwhile, IL-15 treatment can delay or alleviate the senescence of tumor antigen-specific memory CD8^+^ T cells via JAK3-STAT5 signaling activation, reducing the gene expression involved in DNA damage [217]. The new generation of CAR-T cells, which overexpress IL-2 receptor β-chain and a STAT3-binding motif, display enhanced proliferative capacity and a less differentiated phenotype, leading to superior antitumor effects [218]. Indeed, common γ receptor-dependent cytokines and their JAK-STAT pathways are essential in T-cell immunity. However, hyperactivation of the JAK-STAT pathway has been reported only in T-cell lymphomas and not in the literature related to T-cell senescence [219].

Additionally, MAPK and STAT signaling participates in lipid metabolism in senescent T cells. Group IVA cytosolic phospholipase A2 (cPLA2α) is a key molecule that mediates lipid drop formation in T cells. Blockage of p38 and ERK or STAT1 and STAT3 decreases the expression of cPLA2α and the following metabolism reprogramming, eventually inhibiting the Treg-induced senescence of effector T cells [149]. Moreover, inhibition of cPLA2α enhances the anti-tumor immunity mediated by T cells in several mouse models [149].

Notably, the proper function of T cells partially relies on MAPK signaling. Therefore, the appropriate duration of MAPK inhibitor treatment must be determined before therapeutic application to maintain normal immune system function while preventing T-cell senescence and dysfunction in vivo.

### 8.7. Nutrient supplementation

Immunotherapy research seeks to optimize the impact of undernourishment on vaccine efficacy in malnourished, particularly nutritionally deficient individuals. Studies have demonstrated that nutrient supplementation, such as the addition of glucose, is also effective in preventing Treg-induced senescence [119]. Furthermore, senolytic effects and elevated telomere length in T cells can be induced by hyperbaric oxygen therapy, according to a clinical trial [220].

### 8.8. CMV infection prevention

The use of ganciclovir is widespread in preventing and treating CMV infections in patients with a vulnerable immune system and is strongly linked to T-cell senescence. IR induces senescence of splenocyte populations, including T cells, whereas treatment with ganciclovir partially reverses the T-cell proliferative disorder, with reduced p16INK4a and SASP expression [221]; nevertheless, the mechanism necessitates additional research to be fully comprehended.

### 8.9. Exercise

From the activation of mitochondrial function to the regulation of essential signaling pathways, such as NRF2, AMPK, and MAPK [222], the potential health benefits of exercise transcend maintaining and building muscle strength to improving respiratory function, protecting the cardiovascular system, regulating immune function and metabolism, slowing neurological and cognitive decline, and mitigating the chances of diseases associated with aging [223]. Physical activity increases blood oxygen levels and prevents senescence in circulating white blood cells [224]. Older adults who engage in moderate-intensity exercise training may experience an increase in T-cell proliferation, a decrease in senescent T-cells, and an improvement in IL-2 production. Additionally, this type of exercise is linked to longer telomeres and a more robust immune response to vaccines and antigens [225]. This suggests that the mechanism by which exercise reduces cellular senescence warrants further research, such as the production of exercise-alternative drugs.

### 8.10. Telomere transfer mechanisms

A recent study revealed that antigen-presenting cells can secrete telomere-containing extracellular vesicles for acquisition by human peripheral blood-derived T cells, extending T-cell lifespan. T cells that acquire telomeres tend to differentiate into central memory cells, similar to stem cells, and develop the ability to sustain the ability to remember pathogens over an extended period, whereas others gradually senesce. Purified extracellular telomeric vesicle preparations delay human T cell senescence in vitro and enhance the long-term immunity of the mouse immune system [226]. However, the telomere transfer mechanism does not function in all T cells, indicating that preventing certain T cells from senescence is impossible. Meanwhile, validation and therapeutic work utilizing telomere transfer mechanisms in humans in vivo are lacking. Overall, this novel finding suggests a means to extend the lifespan of some T cells or the long-term memory of the immune system, preventing the need for re-vaccination or other immunotherapies.

### 8.11. Challenges and considerations for therapies

Among the drugs that have been reported in studies to combat T-cell senescence, Dasatinib, Quercetin, and Rapamycin have completed Phase I or Phase II clinical trials in the anti-aging field [227,228], while other treatments remain in the preclinical experimental stage.

Particularly, attention should be paid to the side effects and ethical issues in future testing and applications, particularly when clinical trials are planned. Except for ATG and anti-CD153 vaccines, none of these therapies target only T cells but are used as pan-antiaging tools with various side effects in other applications. For example, ABT-263 promotes the development and progression of pulmonary hypertension [229]. Moreover, as the differentiation of all T cells is influenced by mTOR, its potential side effects must be evaluated [230], and alternatively, the appropriate time for drug administration needs to be investigated.

However, ATG targets all T cells while potentially triggering CRS and poor immune reconstitution of CD4+ T cells [231,232]. In addition, given that CD153 is linked to mycobacterial clearance [233], patients with a history of or high susceptibility to mycobacterial infection must be particularly aware of the possibility of neutralizing the antibody clearance of positive cells. Notably, as antibodies do not recognize intracellular molecules, their ability to clear senescent cells after vaccination is limited by the presence of a specific surface marker on the target cells. Therefore, ensuring that this surface marker is accurately and specifically highly expressed during senescence is necessary to avoid unnecessary nonspecific killing, which could compromise the original normal function.

The mechanism of telomere transfer is a newly proposed concept, and whether it can be applied as a therapeutic strategy to provent T-cell senescence requires further investigation. Although the above approaches have shown potential in cellular and animal models, additional research and clinical trials are necessary to confirm their safety and efficacy. Additionally, T-cell senescence is a multifaceted biological process that involves various molecular and cellular mechanisms; therefore, therapeutic approaches targeting T-cell senescence may require a combination of strategies to yield optimal results.

## 9. Discussion

As individuals age, their immune systems undergo significant functional and phenotypic changes across all cellular subpopulations. The aging process has been thoroughly researched concerning T cells, a significant population of cellular immunity, focusing on their modification and adjustment. All the variations in the features of aging T cells and phenotypic defects can be categorized under immunosenescence. Although some studies indicated that senescent T cells can gain innate-like functions after losing antigen-specific killing ability, this ability seems like a compensation for the negative consequences of senescence. Moreover, the interaction between senescent T cells and other senescent cells is unexplored.

Increasing evidence suggests that T-cell senescence is linked to the progression of age-related diseases. Senescent T cells have been most commonly studied in relation to cancer, possibly due to the close relationship between immunosenescence and cancer cell immune escape. Considerable research has also been conducted in the context of cardiovascular disease, for which the precise mechanisms underlying the effects of senescent T cells on the disease process have not yet been explored in depth. Nevertheless, a large body of evidence suggests that T-cell senescence is associated with adverse events. Undoubtedly, microenvironmental disturbances in different diseases promote T-cell senescence through various mechanisms, and the low therapeutic efficacy and the poor prognosis observed in certain cases may be due to the stock of senescent T cells. Therefore, therapies targeting T-cell senescence can be a novel strategy for delaying aging and treating age-related diseases.

The accumulation of senescent T cells is regulated by a combination of distinct pathways. A number of drugs have been developed to eradicate senescent cells or mitigate their effects. However, most currently known pharmacologic interventions lack safety validation for long-term treatment due to their side effects or nonspecificity. Pathway interventions and targeted vaccines may not intervene in all senescent T cells. In addition, the specific benefits of anti-aging obtained through safe modalities, such as exercise and diet, still require further validation.

Therefore, the most pressing issue is identifying suitable specific senescent T cell surface markers, particularly membrane proteins, that serve as precise targets for removing senescent cells using vaccines and other therapies. Immunotherapies for removing senescent cells require careful consideration in clinical translation for vaccination against senescent antigenic epitopes owing to their potential irreversible and long-lasting effects.

Several drugs and vaccines have been showcased to eliminate or inhibit T-cell senescence in mice models or in vitro; however, further trials are needed to assess their safety. Among these, reprogramming T cells to reverse metabolic processes predisposing them to senescence is highly promising. Nevertheless, other cellular tissues should not be overlooked when blocking a particular pathway. Boosting immunity during aging through a combined strategy targeting multiple key pathways may yield more optimized results than a single therapeutic strategy. Exercise should become a universal means of maintaining health and slowing aging across all ages. Approaches to new mechanisms of telomere lengthening by T cells also require investigation and may represent a nascent research area.

Finally, research on tissue-resident senescent memory T cells, as well as their role in age-related diseases is lacking. Targeted elimination of senescent T cells may be a promising therapeutic intervention for age-related diseases, however, there is no safe, effective, or long-term means that entirely prevents senescence in most T cells. Therefore, it is necessary to design novel therapeutics capable of maintaining normal T-cell function and inhibiting senescence. Future research will likely continue to focus on identifying specific targets for different senescent T cells to strengthen the intervention of senescence and age-related diseases and further improve various immunotherapies in vivo. Meanwhile, we emphasize the potential of cellular senescence as an anti-tumor and damage response mechanism [234,235]. Any therapeutic approach aimed at reversing or avoiding senescence should consider the potential risk that removal or reprogramming of senescent cells may disrupt normal physiological function. Therefore, it is important to determine the optimal timing and conditions for therapeutic interventions.

## 10. Conclusion

In this review, we summarize the characteristics of senescent T cells, formation mechanisms, relationships with various aging-related diseases, targets, and interventions, and explore the prospects and possibilities of slowing or reversing T cell senescence or removing senescent T cells. T-cell senescence may be a promising direction in future anti-aging and age-related disease research. Mechanisms of translating the current research on T-cell senescence into therapeutic applications and ensuring the safety and reliability of therapeutic approaches to intervene in T-cell senescence require further research and clinical trials.
